# MIR-4507 Targets *TP53* to Facilitate the Malignant Progression of Non‐small‐cell Lung Cancer

**DOI:** 10.7150/jca.60724

**Published:** 2021-09-13

**Authors:** MengYang Zhao, ZiBo Tang, YiJun Wang, JiaoJiao Ding, Ying Guo, Ning Zhang, TianHui Gao

**Affiliations:** 1 Department of Oncology, Henan Provincial People's Hospital, People's Hospital of Zhengzhou University, Zhengzhou, Henan, 450003, China.; 2 Shenzhen People's Hospital, The Second Clinical Medical College of Jinan University, Shenzhen 518020, China.; 3 Cancer Center, Integrated Hospital of Traditional Chinese Medicine, Southern Medical University, Guangzhou, 510315, China.; 4 Department of Medical Imaging, Henan Provincial People's Hospital, People's Hospital of Zhengzhou University, Zhengzhou, Henan, 450003, China.

**Keywords:** miR-4507, migration, non‐small‐cell lung cancer, proliferation, *TP53*

## Abstract

Lung cancer is a serious threat to human health due to its high morbidity and mortality. microRNAs (miRNAs) are involved in the tumorigenesis and progression of lung cancer. In this study, we elucidated the role of miRNA-4507 (miR-4507) in the pathogenesis of non‐small‐cell lung cancer (NSCLC). miR-4507 is found to be upregulated in NSCLC cells (A549, H460). MTT, 5-ethynyl-2'-deoxyuridine (EdU), wound healing, and transwell assays were performed to evaluate NSCLC cell proliferation and migration. The results demonstrated that miR-4507 inhibition significantly decrease the proliferation and migration of NSCLC cells. Subsequently, a luciferase activity assay was conducted to verify the regulation of the predicted gene target of miR-4507, namely, *TP53*. Mechanism experiments show that miR-4507 activates the PI3K/AKT signal. Further, we co-transfected miR-4507 mimics and *TP53* plasmids and found that *TP53* overexpression could recover the effects of miR-4507 mimics on proliferation, migration, and the PI3K/AKT signal activation. These results suggested that miR-4507 targets *TP53* to facilitate the proliferation and migration of lung cancer cells through PI3K/AKT signal and that miR-4507 could serve as a potential target for NSCLC treatment.

## Introduction

Lung cancer is the most common cancer, with the highest tumor incidence and mortality in the world 1-3. In the latest guidelines published by the World Health Organization (WHO) 4, lung cancer is divided into two subtypes according to the histological classification characteristics: small-cell lung cancer (SCLC) and non-small-cell lung cancer (NSCLC). NSCLC accounts for about 85% of the total lung cancer incidence 5, 6. There are three main subtypes of NSCLC: adenocarcinoma, squamous cell carcinoma, and large-cell carcinoma. Although great advances have been made in cancer treatments, including surgery, radiation, chemotherapy, and targeted therapies 7, lung cancer prognosis remains poor due to the presence of locally advanced or widely metastatic lesions in many patients by the time of diagnosis. Thus, exploring the underlying mechanisms of the tumorigenesis of lung cancer is especially important for lung cancer treatment.

MicroRNAs (miRNAs) are highly conserved in different organisms (including invertebrates, vertebrates, and plants), and they participate in many biological processes, such as cell cycle regulation, differentiation, and development 8. miRNAs are also widely involved in the tumorigenesis of cancers, affecting progression in terms of proliferation, migration, and invasion 9-12. In 2014, miRNA-4507 (miR-4507) was first found to affect the three prime untranslated regions (3'UTRs) of pulmonary surfactant-associated protein A1 (*SFTPA1*), which plays a vital role in maintaining normal lung function and in-host defense 13, 14. In this study, miR-4507 was found to be highly expressed in NSCLC tissues, and further experiments demonstrated its role in tumor proliferation and migration. The target genes of miR-4507 were predicted using bioinformatics websites. Our results revealed that miR-4507 is overexpressed in NSCLC and promotes proliferation and migration of NSCLC cells via *TP53*.

The *TP53* gene, which encodes the p53 protein (which has a molecular weight of 53 kDa), is a famous tumor suppressor gene 15, 16. *TP53* mutations occur in over 50% of malignant tumors 17, 18. However, the mutation frequency of *TP53* is different in different tumor types, with the highest frequency in uterine carcinosarcoma 19. In lung adenocarcinoma, the frequency reaches more than 60%. *TP53* is altered in most SCLC cases and in about 50% of NSCLC cases 20. Researchers have found that lung cancer cells experience apoptosis when wild-type *TP53* is re-expressed after *TP53* mutation or deletion, and clinical trials have confirmed the anti-tumor activity, feasibility, and safety of *TP53* gene therapy 21. *TP53* mutations may also affect the effectiveness of radiotherapy 22. Introducing wild-type *TP53* into tumor cells to recover the function of the p53 protein can help rebuild the tumor's sensitivity to radiotherapy and chemotherapy. Correlations between miRNAs and *TP53* in NSCLC have already been reported, yet the relationships between miR-4507 and *TP53* were first found in the present study. Our work further clarifies the mechanism of *TP53* regulation in NSCLC.

## Materials and Methods

### Cell Cultures

Lung cancer cell lines including H1299, H460, SPC-A1, A549 and normal human lung epithelial cell (BEAS-2B) were obtained from the Cancer Research Institute of Zhengzhou University (Zhengzhou, China) and grown in 1640 supplemented with 10% fetal bovine serum (FBS) (Gibco,USA).

### Cell Transfections

The lung cancer cells were transfected with miR-4507 mimics (cuggguugggcugggcuggg) or NC mimics, and miR-4507 inhibitor (cccagcccagcccaacccag) or NC inhibitor, the transfection efficiencies were evaluated by RT-qPCR. Plasmids were purchased from Vigene Biosciences (Shandong, China). Mimics and inhibitors were designed and synthesized by Guangzhou RiboBio (Guangzhou, China). Before transfection, exponentially growing cells were seeded in a cell culture plate or dish (NEST Biotech, China). Plasmids, mimics, and inhibitors were then transfected into cells using Lipofectamine TM 2000 (Invitrogen Biotechnology, Shanghai, China) according to the manufacturer's protocol. Cells were collected 48-72 h after transfection for further experimentation.

### MTT and EdU assays

Cell proliferation capacity was determined by MTT assay. Cells were seeded in 96-well plates at a density of 3,000 cells/well. After incubation, each well was added using MTT (5 mg/mL) (Sigma-Aldrich, MO, USA), and incubated for 4 h. At the end of incubation, supernatants were removed, and dimethyl sulfoxide (Sigma-Aldrich, MO, USA) was added to each well. The absorbance value (OD) of each well was measured at 490 nm. The calculated rates were then used for curve fitting. All assays were independently performed three times. For EdU assay, proliferating NSCLC cells were examined using the Cell-Light EdU Apollo 488 or 567 *In vitro* Imaging Kit (RiboBio).

### Transwell Assay

The transwell (BD Biosciences, NJ, USA) assay was used to test cell migration abilities. Cells were suspended in 100 mL 1640 without serum and seeded into the top chamber of the transwells coated with Matrigel (BD Biosciences, NJ, USA) or left uncoated, and the bottom chambers were filled with 500 mL 1640 supplemented with 10% FBS. The migrated cells were stained with crystal violet and then photographed and quantified by counting the cell numbers in five random fields. All assays were independently performed in triplicate.

### Wound Healing Assay

Cells were seeded and grew in 6-well plates until a confluent monolayer was reached, and scratches (wounding) were created using a pipette tip. Progression of migration was photographed at initiation and 0 and 48h after wounding. All experiments were repeated at least three times.

### *In vivo* tumorigenesis in nude mice

*In vivo* experiments were approved by the Animal Care and Use Committee of Zhengzhou University and were performed in accordance with the National Institute of Health guide for the care and use of laboratory animals. A total of 1×10^6^ logarithmically growing NSCLC cells transfected with miR-4507 or the control (N=5 per group) in 0.1 ml Hank's solution were subcutaneously injected into the mice (BALB/C, nu/nu, 4 weeks-old, male). After 20 days, the mice were sacrificed and tumor tissues were excised and weighed.

### Quantitative Real-Time PCR

Total RNA was extracted using TRIzol reagent (Invitrogen, USA), and cDNA was synthesized using Hiscript reverse transcriptase (Vazyme Biotech, USA) according to manufacturer's instructions. To measure the miRNA expression, real-time PCR was performed using One Step PrimeScript miRNA cDNA synthesis kit and SYBR premix Ex Taq II kit. Glyceraldehyde-3- phosphate dehydrogenase (GAPDH) or U6 small nuclear RNA (snRNA) was used as an endogenous normalization control. The primers are listed below: miR-4507 (cuggguugggcugggcuggg); TP53 (Forward: CCTCAGCATCTTATCCGAGTGG; Reverse: TGGATGGTGGTACAGTCAGAGC).

### Western blot analysis

Western blotting analyses were performed with standard methods. Proteins were separated by 10% SDS-PAGE gels, and blotted onto polyvinylidene difluoride membrane (Millipore, Billerica, MA, USA). The membrane was probed with the specific antibodies (Cell Signaling Technology, USA; p53: #2524; GAPDH: # 5174S), and then with peroxidase-conjugated secondary antibodies. GAPDH were used as a protein loading control. The bands were visualized by eECL Western Blot Kit (CWBIO Technology, Beijing, China). The images were captured with ChemiDocTM CRS+ Molecular Imager (Bio-Rad, Hercules, CA, USA).

### *In situ* hybridization

Lung cancer tissues were obtained from Henan Provincial People's Hospital, People's Hospital of Zhengzhou University. *In situ* hybridization was carried out by Bioscience Ltd. (Guangzhou, China) to examine miR-4507 expression level in 85 pairs of paraffin-embedded lung cancer specimens. DAB substrate kit (Axy bio, Guangzhou, China) was used to detect positive staining. The extent of staining, defined as the percentage of positively staining tumor cells in relation to the whole tissue area, was scored on a scale of 0-4 as follows: 0, <10%; 1, 10-25%; 2, 26-50%; 3, 50-75%; and 4, >75%. The staining intensity was scored as 0-3 (Negative: 0; Weak expression: 1; Positive expression: 2; Strong expression: 3. The sum of the staining intensity and staining extent scores was used as the final staining score for miR-4507. Inclusion criteria: patients with stage 1-4 NSCLC; patients between 18-85 years old; EGFR and ALK mutation status were not considered; measurable lesions. Cases without clinical staging or lost to follow-up were excluded (10 cases), and 75 cases remained for statistical analysis. This study was approved by the Medical Ethics Committee of Zhengzhou University. Written informed consent was signed by each patient prior to surgery.

### Luciferase Reporter Assay

The day before transfection, the cells are trypsinized and plated, and the confluence of the cells on the second day is controlled to be about 70~80%. In the next day, balance the Hieff TransTM liposomal nucleic acid transfection reagent, vector, miR-4507 mimic, miR-4507 inhibitor, and serum-free MEM to 15~25 °C, dilute an appropriate amount of the vector with serum-free MEM medium, leave it at room temperature for 5 minutes, and dilute with the same medium Hieff TransTM liposomal nucleic acid transfection reagent, placed at room temperature for 5 minutes. Vortex the carrier solution gently, and add the diluted Hieff TransTM Liposome Nucleic Acid Transfection Reagent reagent. Leave it at room temperature for 10-25 minutes to form a DNA-Hieff TransTM complex. Add the compound dropwise to the culture plate, shaking while adding, detect the luciferase activity after 48 hours.

Put the room temperature Dual-Glo® Luciferase Reagent working solution into the sample wells, 70ul per well (at the same time set the culture medium to the empty well), put the 96-well plate on the shaker and shake for 10 minutes to ensure the cells are fully lysed. Move all 140 ul total sample volume to the whiteboard (blackboard) to detect the fluorescence value of firefly. Then add the same volume of 70ul Dual-Glo® Stop & Glo Reagent working solution to the sample well, leave it for 10 minutes to complete the fusion reaction (quench the firefly luciferase and start the Renilla luciferase), and proceed Renal fluorescence value measurement. (Detection ratio = Firefly luciferase detection value/Renilla luciferase detection value).

### Statistical Analysis

All the data were analyzed by SPSS 24.0 (SPSS, Chicago, IL, USA). The data are expressed as the means ± SD from at least three independent experiments. Statistical significance was determined using the Student's two-tailed t test for two groups and a parametric generalized linear model with random effects for the MTT assay. Spearman correlation analysis was conducted to detect the correlation between miR-4507 and P53. All statistical tests were two-sided, and a p value of < 0.05 was considered statistically significant. * P < 0.05, ** P < 0.01, and *** P < 0.001.

## Results

### miR-4507 is elevated in lung cancer and is relates to poor prognosis

**Figure 1:**
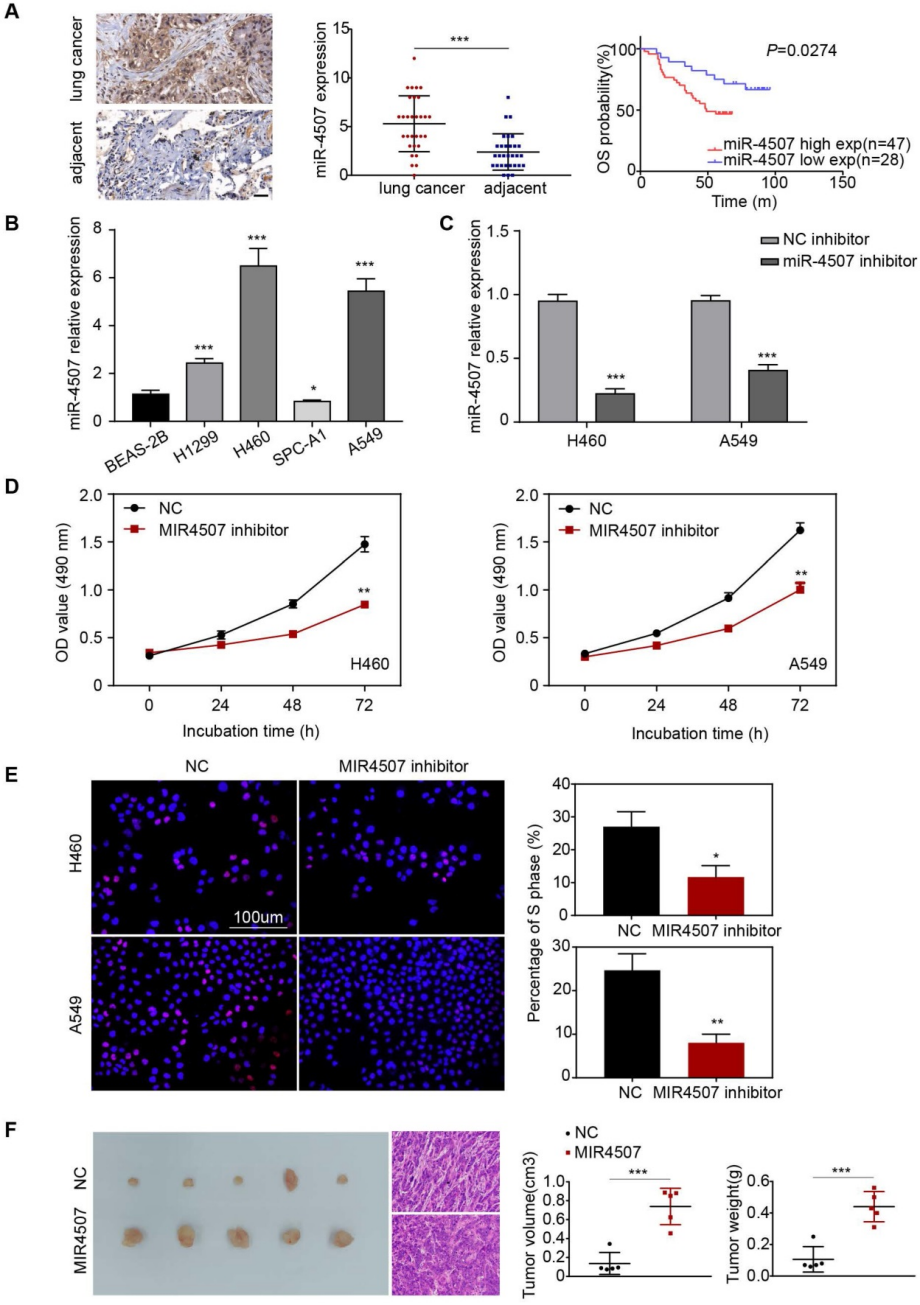
** miR-4507 is elevated in lung cancer and miR-4507 inhibitor suppresses H460 and A549 cell proliferation. (A)** *In situ* hybridization assay was used to detect the miR-4507 expression in lung cancer tissues and adjacent tissues. Scale bar: 50 µm. Kaplan-Meier survival analysis of overall survival of 75 pairs of lung cancer patients based on miR-4507 expression levels (*P*=0.0274). Log-rank test was used to calculate *P* values. **(B)** The expression fold of miR-4507 in BEAS-2B cell and in 4 NSCLC cells (H1299, H460, SPC-A1, A549). The data were shown as the mean ± SD (*P <0.05, *** P <0.001). **(C)** miR-4507 expression levels after transfection with miR-4507 inhibitor. Student's t-test. mean± SD (***P <0.001). **(D)** MTT assays, **(E)** Edu incorporation assays were conducted after transfection with miR-4507 inhibitor or NC inhibitor. Scale bar: 100 µm. **(F)** Xenograft tumors collected on day 20 post subcutaneous implantation of A549-NC, A549-miR-4507 cells on nude mice. Tumor volume and tumor weight were measured on day 20 (n=5), Student's t-test. mean± SD (*P <0.05, **P <0.01, ***P <0.001).

*In situ* hybridization assay was used to detect the expression of miR-4507 in lung cancer patients. Furthermore, survival analysis showed that miR-4507 high expression patients have a poor prognosis compared to low expression patients (*P*=0.0274, Fig. 1A,B), which suggested that miR-4507 serves as an unfavorable factor in lung cancer. Clinical features associated are presented (Table 1). However, no significant correlations were found between miR-4507 expression and patients' age, gender, pathological type or TNM stage.

### miR-4507 downregulation inhibits H460 and A549 cell proliferation

First, we detected the expression level of miR-4507 in different NSCLC cells (H1299, H460, SPC-A1, A549) and in normal human lung epithelial cells (BEAS-2B). miR-4507 was upregulated in NSCLC cells and was especially highly expressed in H460 and A549 cells (Fig. 1B). Next, miR-4507 inhibitors and NC (negative control) inhibitors were separately transfected into H460 and A549 cells, and the transfection efficacy was detected by RT‑qPCR (Reverse Transcription-Polymerase Chain Reaction) (Fig. 1C).

Then, we performed an MTT assay to investigate the effects of miR-4507 on NSCLC cell viability. The results showed that miR-4507 inhibition significantly decreased the viability of NSCLC cells compared to the NC group (Fig. 1D). We further conducted a 5-ethynyl-2'-deoxyuridine (EdU) incorporation assay to detect the effects of miR-4507 inhibitors on NSCLC cell proliferation capacity. The results showed that miR-4507 inhibitors significantly decreased the proliferation capacity compared to the NC group (Fig. 1E).

Further, we performed an *in vivo* tumor formation experiment by subcutaneously injected A549-NC or A549-miR-4507 cells into nude mice. After 20 days, mice injected with A549-miR-4507 had higher tumor burdens than the negative control (Fig. 1F). Statistical analysis showed that the tumor volume and weight of A549-miR-4507 group are bigger than A549-NC group, and is statistically significant (*P*<0.001).

These results demonstrated that miR-4507 downregulation inhibits the proliferation of H460 and A549 cells, and miR-4507 promotes lung cancer growth *in vivo*.

### miR-4507 downregulation inhibits H460 and A549 cell migration

**Figure 2:**
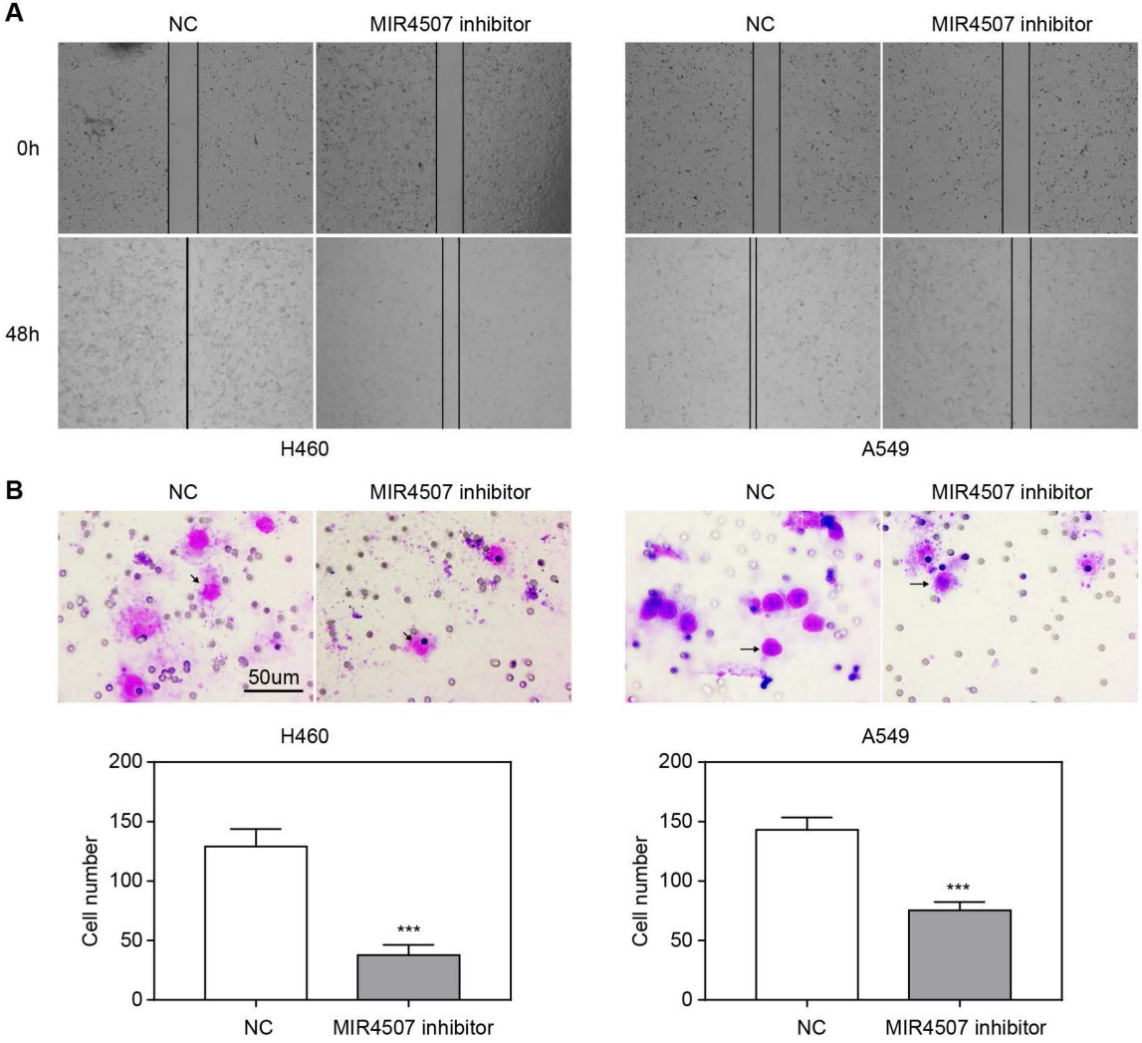
** miR-4507 downregulation inhibits H460 and A549 cell migration.** The effects of miR-4507 inhibitors on NSCLC cell migration capacity were measured by wound healing **(A)** and transwell assays **(B)** in H460 and A459 cells. NC inhibitors were used as negative control. Scale bar: 50 µm. Student's t-test. mean± SD (***P <0.001).

We next explored the effects of miR-4507 inhibitors on NSCLC cell migration capacity by conducting wound healing and transwell assays. The wound area was almost completely healed in the NC group after 48 hours, while it was only slightly reduced in the miR-4507 inhibitor group (Fig. 2A). Further, there were fewer traversing cells in the miR-4507 inhibitor group than in the NC group (Fig. 2B).

These results demonstrated that miR-4507 downregulation inhibits the migration of H460 and A549 cells.

### *TP53* is a direct binding target of miR-4507

**Figure 3:**
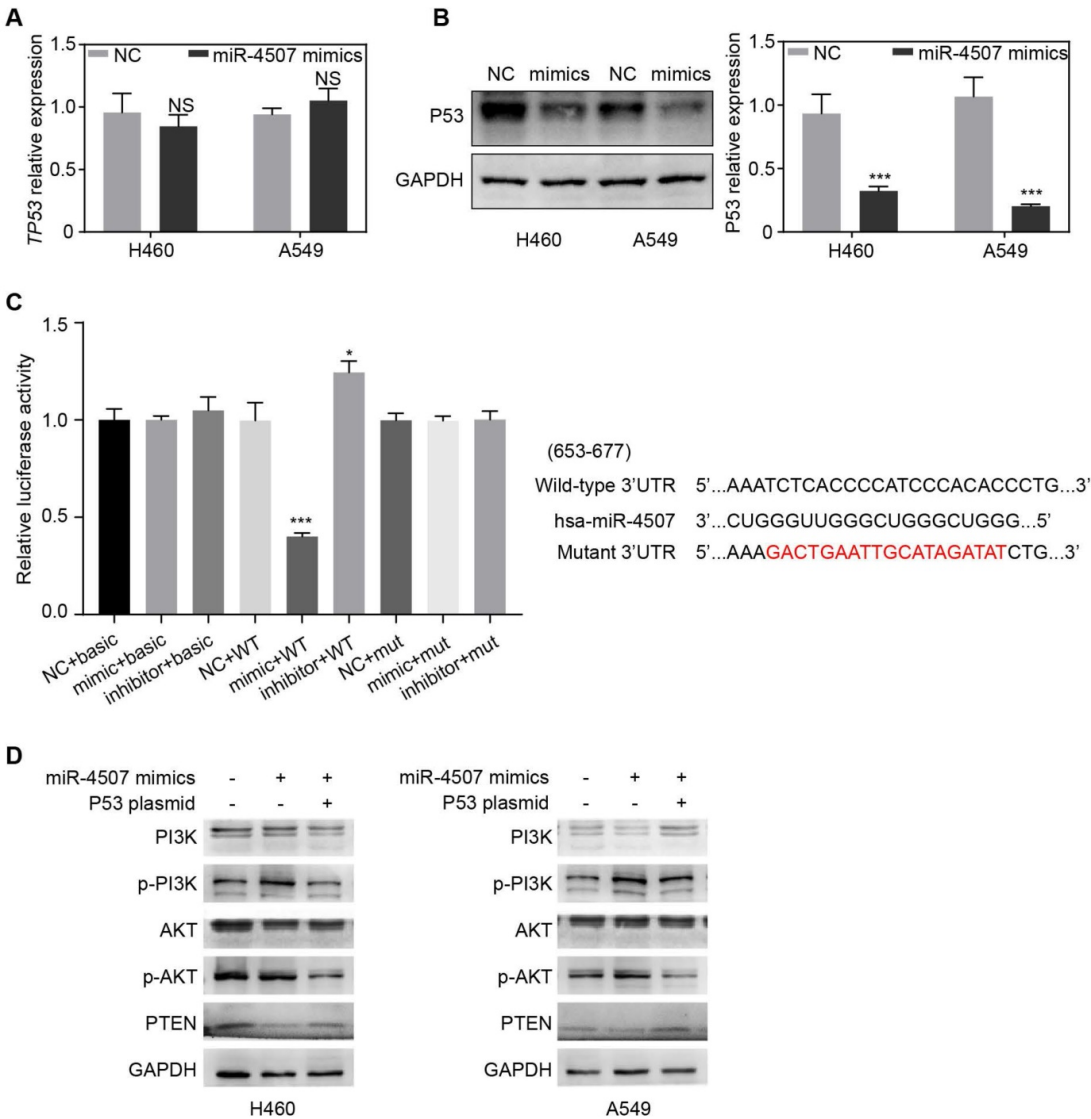
***TP53* is a direct binding target of miR-4507.** *TP53* expression after miR-4507 overexpression in NSCLC cells were detected by RT-qPCR **(A)** and western blot **(B)**, GAPDH was used as a loading control. Student's t-test. mean± SD (NS: no significance, ***P <0.001). **(C)** The luciferase reporter assay was conducted to detect the combination between miR-4507 and 3'UTR region of *TP53* (left lane). Bioinformatics predictions of binding site by miR-4507 in *TP53* 3'-UTR region (right lane). **(D)** Western blot was conducted to detect the expression of p-PI3K/PI3K, p-AKT/AKT and PTEN. Student's t-test. mean± SD (*P <0.05, *** P <0.001).

To investigate the underlying mechanism of miR-4507 in NSCLC cell proliferation and migration, we used the bioinformatic websites miRWalk and miRDB to predict the potential target genes of miR-4507. The screening results indicated that *TP53* was a potential target of miR‑4507. To further clarified the regulatory relations between miR-4507 and TP53, RT-qPCR and western blot were used in both NSCLC cells transfected with NC mimics or miR-4507 mimics separately. RT-qPCR results showed no significant difference of *TP53* expression between miR-4507 mimics and NC group while western blot analysis results showed that miR-4507 mimics downregulated P53 expression (Fig. 3A,B). A dual luciferase reporter assay was conducted to confirm the relationship between miR-4507 and *TP53*. Luciferase activity was reduced in cells co‑transfected with miR-4507 mimics and TP53‑WT (*P*<0.001), and increased in cells co‑transfected with miR-4507 inhibitor and TP53‑WT (*P*<0.05) compared with the NC group. However, the miR-4507 mimics did not change the luciferase activity in cells co-transfected with miR-4507 mimics and TP53‑MT or the cells co-transfected with miR-4507 inhibitor and TP53‑MT (Fig. 3C). These data confirmed that *TP53* is a direct target of miR‑4507.

### miR-4507 activates PI3K/AKT signal through *TP53*

*TP53* and *PTEN* are tumor suppressor which widely involved in the regulation of tumor-related signal pathways. It is well-known that *PTEN* is the negative regulator of the PI3K/AKT signal while it is transcriptionally regulated by *TP53*. Here, we generated the miR-4507 overexpressing cells with/without *TP53* overexpression using miR-4507 mimics and *TP53* plasmid. Western blot results showed that the expression of *PTEN* is downregulated while the PI3K/AKT signal is activated after miR-4507 overexpression in both NSCLC cells. And these effects were rescued with *TP53* overexpression on that basis (Fig. 3D). These results demonstrated that miR-4507 targets *TP53* to regulate the *PTEN*** /**PI3K/AKT signal axis.

### miR‑4507 acts as an onco-miRNA in A549 and H460 cells via the negative regulation of *TP53*

**Figure 4:**
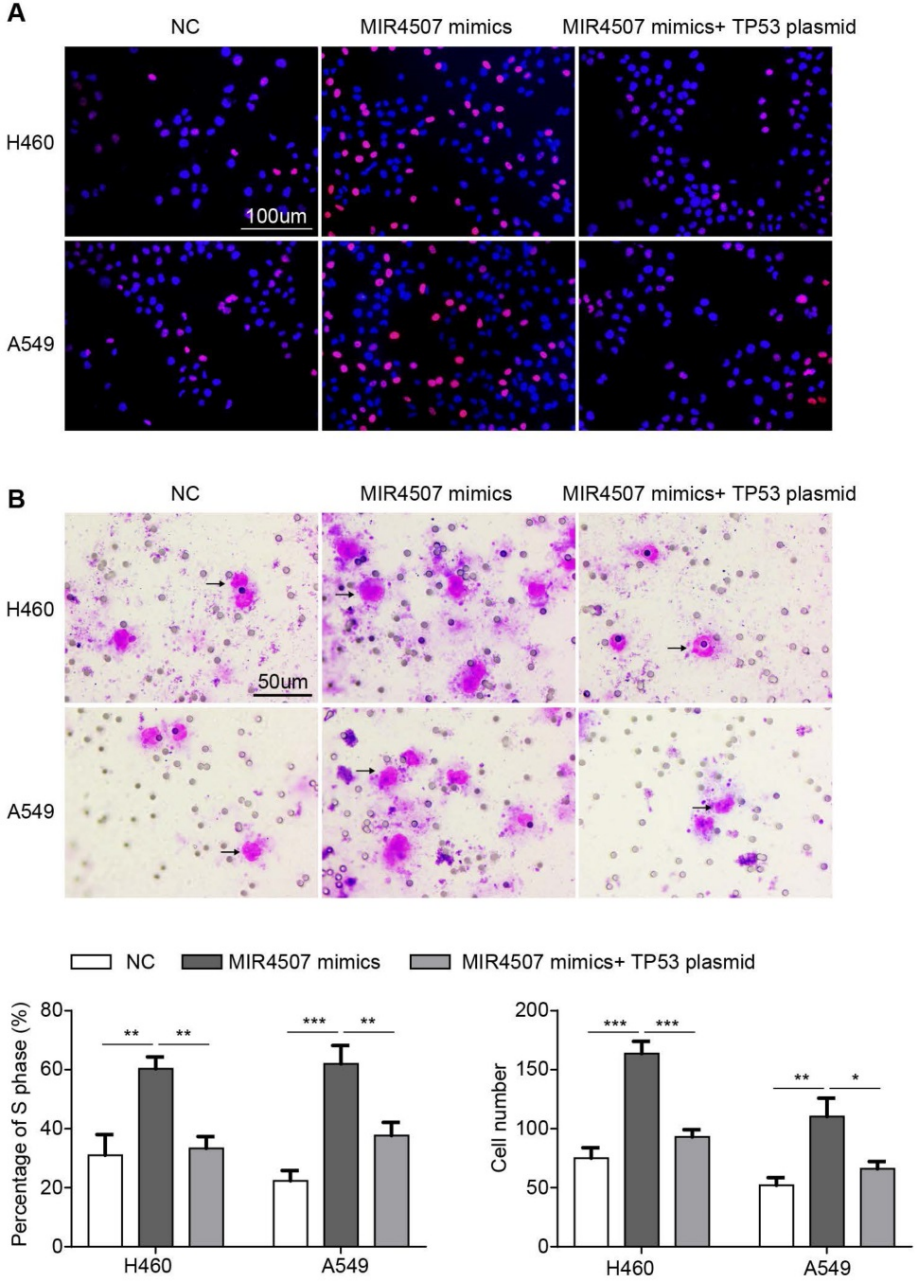
** miR‑4507 acts as a onco-miRNA in A549 and H460 cells via the negative regulation of *TP53.***Edu incorporation assays **(A)** and transwell assays **(B)** were conducted after transfection with miR-4507 mimics with/without *TP53* plasmid. Scale bar: 100µm (Edu), 50 µm (transwell). mean± SD (*P <0.05; ** *P* <0.01, *** *P* <0.001).

To further validate the role of *TP53* in miR-4507 regulation in NSCLC progression, we conducted an EdU assay in three groups separately transfected with NC mimics, miR-4507 mimics, miR-4507 mimics and *TP53* plasmids. The results showed that miR-4507 upregulation promotes proliferation, while this promotion is impeded by *TP53* upregulation (Fig. 4A). Then, a transwell assay was performed to assess the migration capacity in the three aforementioned groups, and the same results were observed; miR-4507 upregulation promotes migration, while this promotion is impeded by *TP53* upregulation (Fig. 4B).

These data indicated that miR-4507 acts as an onco-miRNA in H460 and A549 cells via the negative regulation of *TP53*.

### miR‑4507 expression is negatively correlated with P53 in lung cancer

**Figure 5:**
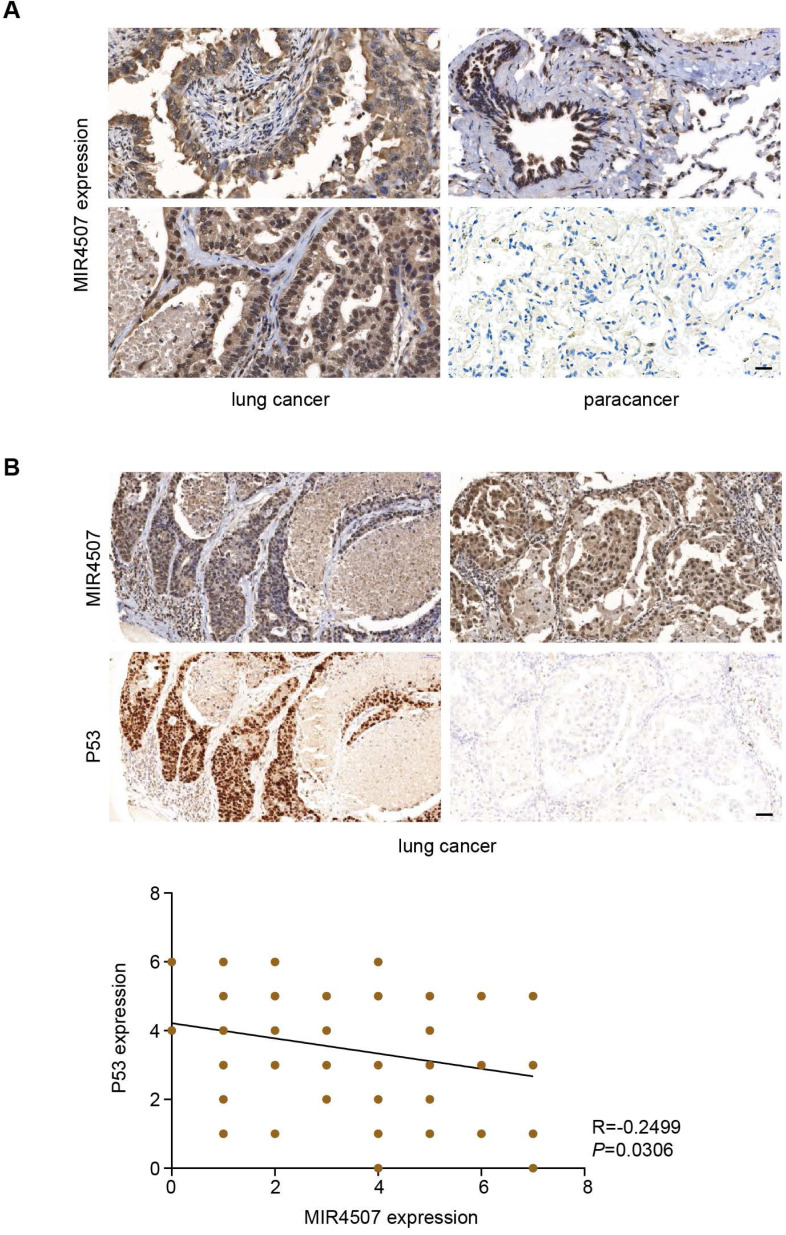
** miR‑4507 is highly expressed in lung cancer tissues and is negatively correlated with P53.** *In situ* hybridization and Immunohistochemistry assays were used to detect miR-4507 **(A-B)** and P53 (B) expression in 75 lung cancer tissues, scale bar: 200 µm. Spearman correlation analysis was conducted to detect the correlation between miR-4507 and P53. Two tailed Spearman correlation analysis. mean± SD (R=-0.2499, *P* =0.0306).

Our study revealed that miR-4507 targets* TP53* to promote the malignant progression of NSCLC, to further demonstrate this regulation on histological level, we used *In situ* hybridization and Immunohistochemistry assay to detect miR-4507 and P53 expression in 75 lung cancer tissues. Our results showed that miR-4507 is highly expressed in lung cancer tissues compared with para cancer tissues (Fig. 5A). Correlation analysis showed that miR‑4507 expression is negatively correlated with P53 expression in lung cancer (R=-0.2499, *P*=0.0306), which proved that our finding has also been confirmed at the histological level (Fig. 5B).

## Discussion

Lung cancer, as one of the most malignant and lethal tumors, is a serious danger to human health. It is characterized by excessive cell proliferation, inevitable distant metastases, and recurrence 23. Abnormal miRNA expression can seriously affect lung cancer progression 24-27. Thus, the exploration of miRNA functions in the pathogenesis of lung cancer is of great significance. miRNAs are involved in every step of tumorigenesis and tumor progression through target genes 28-31. In this study, we found an elevated expression of miR-4507 in NSCLC cells. Subsequently, the NSCLC cell proliferation capacity was determined by MTT and EdU assays in H460 and A549 cells transfected with miR-4507 inhibitors, with the control group transfected with NC inhibitors. The results showed that miR-4507 overexpression significantly promotes cell proliferation. *In vivo* experiments further demonstrated that miR-4507 promotes lung cancer growth. In addition, the NSCLC cell migration capacity was determined by wound healing and transwell assays in H460 and A549 cells transfected with miR-4507 inhibitors, with the control group transfected with NC inhibitors. The results showed that miR-4507 overexpression significantly promotes cell migration. These results indicated that miR-4507 could regulate cell proliferation and migration in NSCLC. However, the related mechanisms need to be studied further.

The miRWalk and miRDB databases were used to predict the target genes of miR-4507. We found that *TP53* is the target gene of miR-4507. Luciferase reporter assay results further demonstrated the hypothesis that *TP53* may be a direct target of miR-4507. Subsequently, *TP53* expression was found to be downregulated in cells with miR-4507 overexpression. These results remind us that miR-4507 may serve as an onco-miRNA in NSCLC through the suppression of *TP53*. *PTEN* was reported to be the downstream effector of *TP53* 32, and it is also the negative regulator of PI3K/AKT signal pathway 33, 34. To fully understand the molecular mechanism of miR-4507 regulation, we overexpressed miR-4507 with/without *TP53* overexpression in NSCLC cells, results showed that miR-4507 activates PI3K/AKT signal by inhibiting *TP53*/*PTEN* expression. Thus, we made a scientific hypothesis that miR-4507 targets *TP53* to activate PI3K/AKT signal and promotes the proliferation and migration of lung cancer cells.

To confirm our inference, we co-transfected miR-4507 mimics with *TP53* plasmids into H460 and A549 cells. Functional analysis results suggested that re-expression of *TP53* in the cells with miR-4507 overexpression recovers the proliferation and migration capacity in both the H460 and A549 cells. Finally, we detected miR-4507 and P53 expression separately in 75 lung cancer tissues, results showed that miR-4507 is highly expressed in lung cancer tissues and is negatively correlated with P53 expression. These results demonstrated our deduction that miR-4507 promotes proliferation and migration through *TP53* in NSCLC cells.

Abnormalities in the P53 signaling pathway are found in most malignant tumors 35-37, and *TP53* mutations are found in over 50% of NSCLC cases^38^, ^39^. Studies have shown that the prognoses of patients with lung cancer with *TP53* mutations are worse than those of patients with wild-type *TP53*, illustrating the importance of *TP53* in lung cancer progression 40. Thus, for patients with NSCLC without *TP53* mutations, it is essential to maintain the physiological function of P53 in their treatment. In this study, two NSCLC cell lines without *TP53* mutations were selected. Additional experiments have revealed the possibility that *TP53* suppression by miR-4507 is an unfavorable factor for patients with NSCLC. This study found for the first time that miR-4507 regulates NSCLC cell proliferation and migration by targeting *TP53*. It may therefore be a potential therapeutic target for patients with NSCLC in the future.

## Figures and Tables

**Figure 6:**
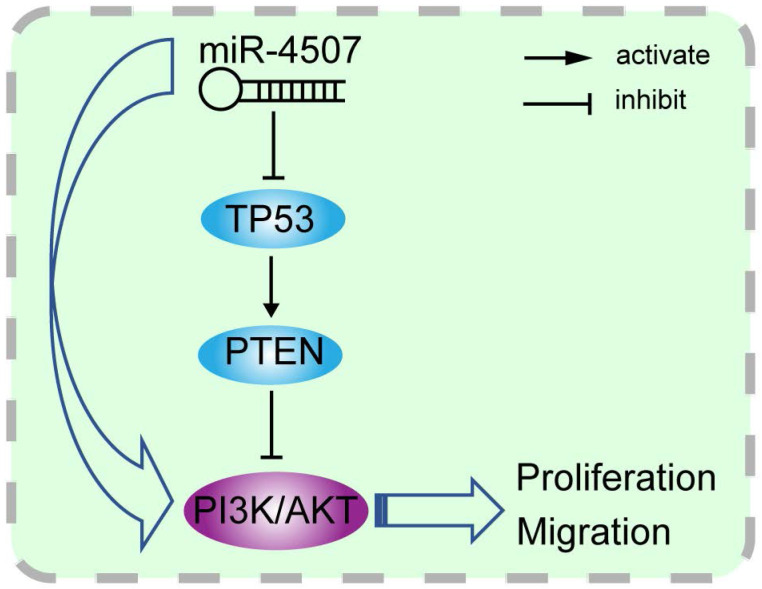
** Illustration of the hypothesised signal mechanism of miR-4507.** miR-4507 targets *TP53* to facilitate the proliferation and migration of lung cancer cells through PI3K/AKT signal.

**Table 1:** Correlation between the clinicopathologic characteristics and expression of miR-4507 in lung cancer patients

Characteristics	n	miR-4507 expression		P value
		High	Low	
**Age, years**				
<60	39	23	16	0.9112
≥60	36	21	15	
**Gender**				
Male	39	24	15	0.7814
Female	36	20	16	
**T stage**				
T1-T2a	58	33	25	0.3792
T2b-T4	17	11	6	
**N stage**				
N0	40	21	19	0.7298
N1-N3	35	23	12	
**M stage**				
M0	71	42	29	0.5485
M1	4	2	2	
**Pathological type**				
Adenocarcinoma	63	40	23	0.1502
Other	12	4	8	

TNM: tumor, node, metastasis. Pathological type: other includes Bronchioloalveolar carcinoma and Mucoepidermoid carcinoma. P values by t test.
